# Biochemical Markers of Bone Turnover in Patients with *β*-Thalassemia Major: A Single Center Study from Southern Pakistan

**DOI:** 10.1155/2016/5437609

**Published:** 2016-02-23

**Authors:** Sadia Sultan, Syed Mohammed Irfan, Syed Ijlal Ahmed

**Affiliations:** ^1^Department of Hematology & Blood Bank, Liaquat National Hospital and Medical College, Karachi, Pakistan; ^2^Liaquat National Medical College, Karachi, Pakistan

## Abstract

*Objectives*. Skeletal complications in *β*-homozygous thalassemic patients are uncommon but often debilitating, even amongst children and adolescent patients with well maintained transfusion and chelation therapy. The aim is to evaluate the biochemical markers of bone turnover in regularly transfused thalassemic patients and its possible correlations with demographic data and hematological and biochemical markers.* Methods*. In this prospective cross-sectional study, 36 *β*-thalassemia major patients were enrolled from March 2012 to March 2014. All patients underwent complete blood counts, LFTs, serum ferritin, serum calcium, phosphorus, serum albumin, alkaline phosphatase, 25-OH vitamin D, and parathormone (PTH) levels.* Results*. There were 17 males and 19 females with mean age of 12.56 ± 5.9 years. Hypocalcemia and hypophosphatemia were seen in 66.6% and 19.4%, respectively, while 25-OH vitamin D deficiency was present in 72.2% of thalassemic children and adolescents. Hypoparathyroidism was seen in 13.8% while hyperparathyroidism was detected in 8.3% of patients. There was direct correlation between serum phosphorus and ferritin levels (*P* < 0.05). No correlation was found between indirect bilirubin and skeletal parameters, calcium and parathyroid hormone (*P* > 0.05).* Conclusions*. Biochemical profile is significantly altered in patients with *β*-thalassemia major and bone associated biochemical abnormalities like hypocalcaemia, 25-OH vitamin D deficiency, and hypophosphatemia are not uncommon in Pakistani patients with thalassemia major.

## 1. Introduction

Thalassemia is among the most common genetic disorders worldwide. *β*-thalassemia is an autosomal recessively inherited hemoglobinopathy which is much prevalent in Pakistan [1]. It is anticipated that 4000 to 9000 newborns with *β*-thalassemia major are being added each year to the existing disease pool. The carrier frequency is 5 to 8% in our part of world and is seen uniformly in all ethnic groups [2].

*β*-thalassemia major is characterized by severe hemolytic anemia that entails regular blood transfusion. Life expectancy of such patients is noticeably prolonged with regular blood transfusion and appropriate iron chelation conversely; consequently, secondary hemochromatosis is still a major challenge.

In thalassemic patients, bone disease is an important cause of morbidity. Various skeletal complications including osteopenia, osteoporosis, scoliosis, rickets, spinal deformities, nerve compression, and spontaneous fractures are regularly reported in transfusion dependent thalassemics [3, 4]. Diverse factors that contribute to skeletal disease including medullary expansion, iron accumulation, abnormal calcium-phosphorus balance, high bone turnover, hormonal insufficiency, and lastly hypoxia may influence skeletal complications [5].

Deranged calcium homeostasis is a consequence of iron accumulation in *β*-thalassemic patients. Additionally, the defective synthesis of 25-OH vitamin D and hypoparathyroidism has also been frequently reported in these patients, which have negatively affected their bone metabolism.

Our aim is to estimate the biochemical markers of bone turnover in children and adolescent thalassemic patients and to examine the association of abnormal profile with demographic features and hematological and biochemical markers at a tertiary care center in Southern Pakistan.

## 2. Materials and Methods

There was a prospective cross-sectional study extended from March 2012 to March 2014. 36 patients with *β*-thalassemia major were enrolled. An informed consent was obtained from parents/guardians in patients aged <18 years and from patients aged ≥18 years.

Demographic data including age, gender, and medical history was recorded by thorough history from parents/guardians and from patient's medical record. Hematological parameters including hemoglobin/hematocrit, WBC, and platelets were determined by automated Cell Dyne Ruby counter (Abbott, USA). Liver function test, serum calcium, and phosphorus were evaluated on Hitachi 912 instrument through photometric assay except for serum ferritin which was measured by immunoturbidity methodology. Serum parathormone was detected by Cobas e 411 analyzer (Roche, Japan) by chemiluminescence technique.

Patients were stratified into two groups, age of ≤ 15 years and age of >15 years, to determine the possible bone profile distinction in relation to age.

The research protocol was approved by the institutional thical and research committee of Liaquat National Hospital prior to the study.

### 2.1. Data Analysis

A statistical analysis was carried out using IBM statistics SPSS version 21. Results were reported as the mean (SD) and independent sample *t*-tests were used to compare study groups. The descriptive statistics of bone mineral profile were measured and their correlations were calculated. Comparison of categorical data was carried by Chi-square test. *P* value of <0.05 was considered as statistically significant.

## 3. Results

### 3.1. Demographic Profile

The study included 36 homozygous TM patients with the mean age of 12.56 ± 5.9 (5–24) years. 17 were males and 19 were female with the mean age of 15.5 ± 5.9 and 10.1 ± 4.5 years, respectively. The descriptive statistics and laboratory parameters are shown in Table 1.

### 3.2. Hematological and Biochemical Findings

The mean hemoglobin level was 7.4 ± 1.9 gm/dL with the hematocrit of 23.1 ± 5.7%. The mean total leukocytic count was 6.5 ± 3.9 × 10^9^/L and mean platelets count was 150.7 ± 94.1 × 10^9^/L. Serum ferritin levels were found to be markedly elevated 4699.7 ± 3089 ng/mL. Total bilirubin and direct and indirect bilirubins were 1.53 ± 0.8, 0.56 ± 0.5, and 0.94 ± 0.5 mg%, respectively. Serum ALT and AST were 78.5 ± 73.7 and 68.0 ± 42.7 U/L, respectively.

### 3.3. Biochemical Profile

The mean corrected calcium, phosphorus, and serum 25-OH vitamin D levels were found to be 8.1 (±0.8), 3.1 (±1.28), and 23.1 (±10.7), respectively. The mean parathyroid level was 23.5 (±23.0). Hypocalcemia and hypophosphatemia were seen in 66.6% and 19.4%, respectively, while 25-OH vitamin D deficiency was present in 72.2% of thalassemic patients. Parathormone deficiency was seen in 13.8%, while hyperparathyroidism was detected in 8.3% of patients.

**Figure 1:**
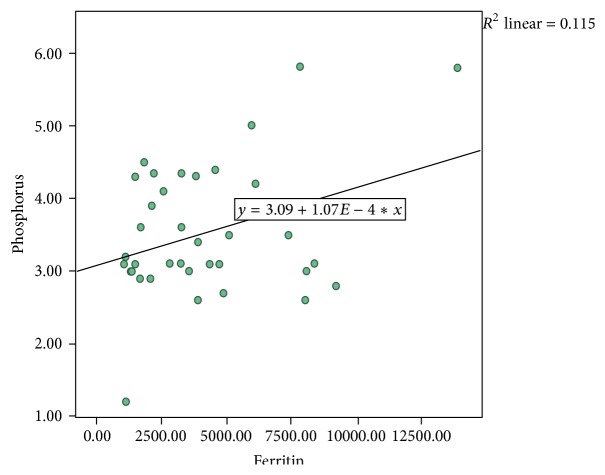
Correlation of serum ferritin and phosphorus (*P* = 0.04).

The correlations were also calculated among variables. There was positive correlation between low serum phosphorus and high ferritin levels (*P* = 0.04) as shown in Figure 1. No correlation was found between indirect bilirubin and skeletal parameters, calcium, and parathyroid hormone (*P* > 0.05). No statistically significant difference was noted in two stratified age groups with respect to testing parameters.

## 4. Discussion

The advent of safe transfusions with adjuvant chelation therapy has drastically prolonged the life expectancy in patients with *β*-thalassemia major. However, this brought various nonsiderotic, hematological, biochemical, and systemic complications. A sequence of skeletal complications including osteopenia, osteoporosis, scoliosis, skeletal deformities, bone pain, nerve compression, and spontaneous pathological fractures is not infrequent in transfusion dependent thalassemics [3, 4].

The anterior pituitary is principally vulnerable to iron accumulation which interrupts hormonal emission, leading to various endocrine dysfunctions. These endocrinological manifestations include hypothyroidism, hypoparathyroidism or growth failure, and gonadal damage. Hypoparathyroidism being assessed by parathormone levels, serum calcium, and phosphate has been reported in various studies with a prevalence of 4–40% [6, 7]. The prevalence of hypoparathyroidism (13.8%) in our study was analogous to several studies from various countries like Iran (7.6% to 14.6%), Oman (19%), China (10.7%), and Greece (13.5%) [8–12]. Nonetheless, a retrospective study from Pakistan found a higher prevalence of hypoparathyroidism (40%) among *β*-thalassemic patients [7]. The plausible explanation for this variation in prevalence of hypoparathyroidism could be attributed to small sample size (*n* = 10) and might be due to the retrospective nature of that study. One finding is the negative correlation of parathormone with indirect bilirubin determined in the present study but statistically not significant. Possibly, both deranged unconjugated bilirubin and low parathormone are sequel of hemosiderosis.

Parathormone is mainly responsible for the regulation of calcium homeostasis in body. Hypoparathyroidism can lead to hypocalcemia and osteoporosis [4]. Calcium is a very important mineral in human body playing a major role in skeletal mineralization [13]. Phosphorous has predominant effect in combination with calcium on bone growth and development, whereas osseous tissue constitutes 85% of total body phosphorus [4]. Results of the present study showed diminished level of corrected serum calcium and inorganic phosphorus in *β*-thalassemic patients. These findings are consistent with those of Adil et al. and Mirhosseini et al. [7, 14].

The serum corrected calcium level was considerably low in our patients. Our results were in concurrence with Shamshirsaz et al., Aleem et al., Zamboni et al., and Vogiatzi et al., who also found significant hypocalcaemia in their thalassemic patients [9, 15–17]. The main explanation of hypocalcaemia is endocrinopathy secondary to hemosiderosis. However, chelation therapy in addition to advanced liver diseases may also contribute to hypocalcemia. On the contrary, some authors found no significant difference between the patients and controls in serum calcium level [18, 19].

In relation to inorganic phosphorous level, we found that majority had normal levels; only 19.4% had hypophosphatemia. The possible explanation of hypophosphatemia is secondary to hyperparathyroidism (8.3%) in our series. These findings were analogous to studies that reported that phosphorous levels were within the normal range in patients compared to controls [20]. On the contrary, some researchers reported significantly higher serum phosphorous levels in thalassemic patients than the control groups [21, 22]. In the present study, low serum phosphorus positively correlated with high ferritin, but we could not ascertain its significance in our thalassemic patients.

We also determined that low calcium is associated with raised indirect bilirubin levels (*P* > 0.05). In normal individuals, calcium and bilirubin can react to form calcium bilirubinate [23]. Serum calcium complexes with indirect bilirubin and thereby decreases the concentration of serum calcium levels [23].

Thalassemic patients show marked deficiency (72.2%) of 25-hydroxy vitamin D in our study. Napoli et al. found that 9.6% of thalassemic patients had vitamin D insufficiency in Italian thalassemics [24]. Low serum 25-hydroxy vitamin D levels have also been reported previously in *β*-thalassemic patients by many investigators [25–27]. This deficiency has been attributed to malabsorption of vitamin D as well as inadequate dietary intake [28–30]. Another plausible explanation is hepatic dysfunctions which lead to defective hydroxylation of vitamin D resulting in decreased level [31]. Other authors also reported that the etiology of 25-OH-D deficiency might be the hepatic iron-overload rather than the dysfunctioning of endocrine tissues [32].

In conclusion, BTM patients have markedly deranged biochemical profile. Aggressive nutritional support and calcium/vitamin D supplementation are highly recommended for these patients. Regular monitoring of biochemical mineral profile is also recommended. Proper monitoring and treatment will definitely improve skeletal status in these patients.

## Figures and Tables

**Table 1:** Descriptive statistics and laboratory parameters.

Parameters	Results	References ranges
Age (years)	12.56 ± 5.9	—
Hemoglobin (gm/dL)	7.4 ± 1.9	12–16 gm/dL
Hematocrit (%)	23.1 ± 5.7	36–48
Total leucocyte count	6.5 ± 3.9	4.0–11.0 × 10^9^/L
Platelet count	150.7 ± 94.l	150–400 × 10^9^/L
Total bilirubin (mg/dL)	1.53 ± 0.8	0.3–1.2
Direct bilirubin (mg/dL)	0.56 ± 0.5	0.1–0.5
Indirect bilirubin (mg/dL)	0.94 ± 0.5	0.2–0.7
AST (*μ*/L)	68.0 ± 42.7	<40
ALT (*μ*/L)	78.5 ± 73.7	7–56
Serum ferritin (ng/mL)	4699.7 ± 3089	20–200
Corrected serum calcium (mg/dL)	8.1 ± 0.8	8.6–10.2
Serum phosphorus (mg/dL)	3.1 ± 1.28	2.5–4.5
Serum vitamin D (ng/mL)	23.1 ± 10.7	>30
Parathyroid level (pg/mL)	23.5 ± 23.0	12–50
